# An exploratory survey of money boys and HIV transmission risk in Jilin Province, PR China

**DOI:** 10.1186/1742-6405-7-17

**Published:** 2010-06-17

**Authors:** Xiangdong Meng, Allen F Anderson, Lu Wang, Zhihe Li, Wei Guo, Zixuan Lee, Huixin Jin, Yong Cai

**Affiliations:** 1 Jilin Provincial Center for Disease Prevention and Control, Changchun, China; 2 Indiana University South Bend, South Bend, Indiana, USA; 3 National Center for STD & AIDS Prevention and Control, Beijing, China; 4 The Institute for Dermatosis Prevention and Control, Harbin, China; 5 Jilin Municipal Center for Disease Prevention and Control, Changchun, China

## Abstract

This report represents the first exploratory study of Chinese men who provide commercial sex services to other men ("money boys") in Jilin Province, People's Republic of China, through a convenience sample drawn from Changchun and Jilin City. A total of 86 active money boy participants (Changchun, n = 49; Jilin City, n = 37) were surveyed concerning background and demographics, basic HIV transmission knowledge, and sexual practices. The survey indicated that while Jilin Province money boy behavior matches other studies concerning propensity to high risk behavior and significant bridging potential, the Jilin money boys, unlike previous studies, exhibited a high level of basic HIV/AIDS transmission knowledge. In spite of this level of knowledge, none of the participants reported always using a condom in their sexual activities. They also exhibited a high level of awareness of voluntary counseling and testing available in the province, yet relatively few had availed themselves of these services. These preliminary findings will be used as a baseline and springboard for continuing study in the Jilin Province money boy community. Even now, however, it is becoming clear that the dynamics of male commercial sex work may vary greatly depending upon local influences, and will necessitate that future interventions are highly tailored to area-specific circumstances.

## Introduction

The dire progression of the Human Immunodeficiency Virus (HIV) in China was highlighted in early 2009, when reports emerged that it was the leading cause of infectious disease mortality during the previous year [1]. While the national prevalence is estimated to be only 0.1% [2], the epidemic is driven by high-risk behaviors within particular sub-populations that create pockets of high infection. These pockets often serve, in turn, as conduits for the disease into the broader heterosexual population through complex behavioral dynamics (i.e., bridging activities related to sex work and drug use, etc.) that are yet to be fully investigated [3-6].

One such area of high prevalence is among men who have sex with men (MSM). While much effort was placed early-on into understanding the nuances of HIV transmission in the drug injector and female sex work populations, general recognition of the depth and breadth of the MSM population was not acknowledged. It was only after 2003 that the MSM population began to receive broad scrutiny on the mainland; indeed, it was not until 2002 that the MSM population was included in the national HIV sentinel surveillance [7,8]. UNAIDS now reports that the HIV prevalence within the Chinese MSM population is within a range from 2.5-6.5 percent [9].

Under Confucianism, the traditional family unit is patriarchic, with a dedication to the family line and filial piety. Confucius viewed the husband-wife relationship as one of the five primary human relationships that shape society [10]. Such cultural forces have necessitated that homosexual conduct be highly cloistered. Fear of ostracism has even driven many homosexuals to marry in an attempt to conform socially and satisfy family pressures and expectations [11,12].

No subgroup within this population is more secluded than the so-called "money boys" (MBs), or males who provide commercial sex services to other males. In addition to the socio-cultural dynamics mentioned above, male commercial sex work is now prosecuted under the crime of prostitution, which was formerly viewed in the law as an exclusively female offense [13,14]. The resulting need for covertness has prevented systematic study of the MB phenomenon until relatively recently.

Early research suggests that the risk taking in the broader MSM community is magnified among money boys. Generally, the research indicates that MBs "rarely insist on condom use, and may engage in unprotected anal sex because they are following the wishes of their clients [12,15-17]." Only one study, to date, has found that rural money boys may increase condom usage after migrating to large cities [17]. The very nature of the MB trade would imply that they seek multiple sexual partners, and may yield to client wishes not to use safer sex practices. Researchers have also found that money boys generally have "substantial misconceptions regarding HIV transmission, prevention, diagnosis, and treatment [18]." Non-transmission modes seem particularly problematic, with many believing that the virus could be acquired through mosquito bites, shaking hands, or eating with an infected individual. In addition to significant misunderstanding about HIV transmission, it is becoming apparent that many MBs are not aware of the voluntary counseling and testing (VCT) services that are now available in many parts of China [3,18,19]. The picture that is emerging of MB behavior includes high risk taking, multiple intimate partners, minimal understanding of virus transmission, the potential for bridging, and a mix awareness and use of VCT. It is clear that the intuitive dangers from MB activity that have been speculated upon now have growing empirical support.

The present report discusses the first exploratory research excursion into the money boy phenomenon in Jilin Province in northeastern China. The tantalizing seminal findings that emerged from this first look are preliminary, and will serve as a baseline and springboard for future analysis.

## Methods

After institutional ethical approval was obtained, participants (N = 86) for the present investigation were recruited to form a convenience sample from the Changchun (n = 49) and Jilin City (n = 37) areas, the two largest cities in Jilin Province. Changchun has an approximate population of 4 million within the city limits, and Jilin City's population is around 1.9 million. By Chinese standards, these are not exceptionally large population centers. As such, they offer a different setting from previous MB studies [3,6,12,18,19], with were conducted in very large and/or highly westernized metropolitan centers.

A money boy was defined as any male who had provided sex services (receptive and/or penetrative) to other males for a fee within the previous six months. Given the extremely covert nature of such activity in Jilin Province, initial access into the population was gained through a relationship being established between the research team and MSM non-governmental organizations (NGO) in each city. Actual participant contact was accomplished through a total of six facilitators who were MSMs associated with the respective non-governmental organizations. The use of NGOs to facilitate contact with money boys has been employed in several recent studies [15,18,19]. This approach is a variant of Community Identification Process sampling [20], used to identify hard to reach populations, and was employed by Choi, et al. [21] in their Beijing MSM study. Facilitators were trained to make appropriate contact with MBs and seek their voluntary participation in the survey. Four facilitators were utilized in Changchun, and two in Jilin City. Each of these individuals facilitated successful contact with 12-16 money boys.

Once agreement to participate had been achieved by the six facilitators, two trained professionals from the research team made contact with the individuals and, after informed consent, administered a limited and structured face-to-face interview with each MB. After arrangement by the contact facilitator, no money boy refused to be interviewed by representatives of the research team. Interviews were conducted in October-November, 2008, and took place privately in bath houses and gay activity clubs. On average, interviews lasted 15 minutes. Interviewees received no financial incentive for their participation. The interview contained queries on background and demographics, basic HIV transmission knowledge, and sexual practices, with the previous six months as the time reference for behaviors. Questions were limited and basic so as not to make the interviewees uncomfortable or reticent at this early juncture of our work with them.

## Results

### Demographics

The 86 participants (Table 1) ranged in age from 17 to 38, with a mean age of 24.6 (SD = 3.22). Sixty-eight (79.1%) were between 20 and 30 years of age. While the majority (74.4%) was single, nearly one quarter (24.4%) were married. One additional participant (1.2%) reported being divorced.

**Table 1:** Demographics (N = 86)*

Variable	Number	Percent
Age		
<20	4	4.65
20-30	68	79.07
31-40	14	16.28
		
Marital Status		
Single	64	74.42
Married	21	24.42
Divorced	1	1.16
		
Registered Residence		
Urban	56	65.10
Rural	30	34.90
		
Employment (n = 84)		
Full-time	38	44.19
Part-time	40	46.51
Unemployed	6	6.68
		
Education		
≤Middle School 33	38.37	
High School	39	45.35
College	14	16.28
		
Monthly Income (yuan)		
<1000	24	27.91
1000-2000	43	50.00
2000-3000	12	13.95
>3000	7	8.14
		
Present Residence		
City	56	65.12
County	22	25.58
Countryside	8	9.30

*Unless otherwise specified.

Thirty-three (38.4%) of the MBs had a middle school or less education, while 53 (61.6%) had graduated from high school or college. Seventy-eight (90.7%) were employed elsewhere either full-time or part-time. Only 6 (7.0%) of the participants reported being unemployed. Slightly over one quarter (27.9%) made less than 1000 RMB (approximately $143.00US) per month at their place of employment; yet, 62 (72.1%) made greater than 1000 RMB per month, and 19 (22.1%) made over 2000 RMB (approximately $286/00US) per month.

The majority of participating MBs (79.1%) were registered residents of Jilin Province. This comparatively small number of individuals from other provinces may be a product of, *inter alia*, the relative level of employment opportunities for migrants in the province, as well as the bitter cold winter conditions. Even when broken into urban and rural categories, those registered in either Changchun or Jilin City (n = 56) outnumbered those registered in rural areas (n = 30).

### Sexual Practice

Sixty-eight (79.1%) of the participants self-identified as homosexual, while 18 (20.9%) reported a bisexual orientation (Table 2). None of the 86 participants reported being heterosexual. Sixty-one (70.9%) of the MBs admitted having commercial sex within thirty days of their respective interview. Anecdotally, the six seeds contended that they had personal knowledge that all participants had engaged in commercial sexual activity during the previous month. As hearsay, this had no impact in the analysis.

**Table 2:** Commercial Sexual Behavior (N = 86)*

Variable	Number	Percent
Self-Identified Sexual		
Orientation		
Homosexual	68	79.07
Bisexual	18	20.93
Heterosexual	0	00.00
Commercial Sex Activity		
Within the Last Thirty Days		
Yes	61	70.93
No	25	29.07
CondomUse		
Always	0	00.00
Sporadic	56	65.11
Never	30	34.88
Condom Use During Last		
Anal Receptive Experience		
Yes	54	62.79
No	36	37.21
Condom Use During Last		
Oral Receptive Experience		
Yes	23	26.74
No	63	73.26
Natureof Commercial		
Sexual Contact		
One exclusive client	24	27.90
Only random clients	31	36.05
Both random and exclusive 31	36.05	
Locationof Commercial		
Sexual Contact (n = 82)		
Private House	24	29.26
Public Bath or Hotel	43	52.43
Both Private House or Public 15	18.29	
Venue		
VCT Service Awareness		
Expressed Awareness 68	79.07	
Unaware	18	10.93
VCT Service Use		
Used	14	20.00
Notused	72	80.00

*Unlessotherwise specified.

While level of commercial sexual activity, reported in an open-ended format, varied widely, 29 (33.7%) of the MBs acknowledged greater than 20 total sexual partners. Below this level of activity, total sexual partners ranged from 1 to 13, with 4 being the mean number. Twenty-four (27.9%) reported only one regular commercial sexual partner, while 31 (36.1%) had both a regular commercial partner as well as more than one more random (i.e., single encounter or highly sporadic encounter) commercial partner, and 31 (36.1%) had only random commercial partners. Place of commercial sex activity included private houses, bathhouse rooms, and hotel rooms, with those who had only one regular commercial sexual partner typically meeting in private houses.

None of the 86 MBs reported "always" using a condom in their sexual activities over the past six months. Only 56 (65.1%) reported using condoms during that time period, leaving 30 (34.9%) who never used condoms. Fifty-four (62.8%) had used a condom during their last anal-receptive contact, and 23 (26.7%) during their last oral-receptive contact. There was no difference in condom usage between those that solicited clients from bars and bath houses and those who used the internet.

Significantly, 18 (20.9%) had also employed a female commercial sex worker. This number directly corresponds to the number of participants who self-reported being bisexual. As well, though probably not mutually exclusive, the 21 participants who are married are likely to be maintaining some degree of sexual relationship with their spouse.

### HIV/AIDS Knowledge

The interview instrument included eight questions related to each participant's level of virus transmission knowledge (Table 3). If an individual scored six or more correct answers, his level of understanding was classified as "good." Seventy (81.4%) MBs scored in this good understanding category. In spite of this relatively high level of awareness relative to other MSM/MB studies, 46 (65.7%) of these 70 money boys reported frequent unprotected sexual activity. As well, it must be remembered that none of the MBs "always" used a condom, yet 79 (91.9%) responded correctly to the specific question on condom use (Table 3, Question 7). Most participants reported that they simply did not receive the same physical and psychological contentment when using a condom.

**Table 3:** Basic HIV/AIDS Transmission Knowledge (N = 86)

Transmission Knowledge Query	Response	Percentage
1. Can you identify an HIV-positive Person by his/her appearance		
Yes	11	12.79
No	63	73.26
Don'tKnow	12	13.95
		
2. Can HIV be transmitted through a mosquito bite?		
Yes	16	18.60
No	59	68.61
Don't Know	11	12.79
		
3. Can you contract the virus by eating with an HIV-positive person?		
Yes	5	5.81
No	77	89.53
Don't Know	4	4.65
		
4. Can the virus be contracted through a blood transfusion?		
Yes	84	97.67
No	0	00.00
Don't Know	2	2.33
		
5. Can the virus be contracted through sharing injecting equipment?		
Yes	78	90.70
No	3	3.49
Don't Know	5	5.81
		
6. Can an HIV-positive pregnant female transmit the virus to her fetus?		
Yes	81	94.18
No	3	3.49
Don't Know	2	2.33
		
7. Does the correct use of a condom reduce the probability of HIV transmission?		
Yes	79	91.86
No	3	3.49
Don't Know	4	4.65
		
8. Can having one exclusive sexual partner reduce the probability of HIV transmission?		
Yes	71	82.56
No	8	9.30
Don't Know	7	8.14

Continuing their relatively high level of information about HIV/AIDS, 68 (79.1%) of the sample knew of the government's voluntary counseling and testing program. Again, however, this high level of awareness did not translate into actual utilization. Only 14 of the 70 participants who exhibited "good" HIV/AIDS knowledge had used the country's widespread counseling and testing services. None of those scoring less than "good" on the transmission knowledge queries had sought VCT services. Primary rationalizations stated by the participants for under-utilization were the anticipated mental stress of being found HIV-positive and the social stigma that the status carries on the mainland.

### Bivariate Analysis

Participants proved quite homogenous concerning knowledge and behavior whether residing in Changchun or Jilin City; that is, no statistically significant difference (requiring P < 0.05) was found between the two locations. However, significant differences emerged when the group was analyzed from the urban (n = 56) and rural (n = 30) perspective. While 49 (87.5%) of the urban registered MBs scored "good" or better on HIV/AIDS knowledge, 21 (70.0%) of the rural residents scored the same (X^2 ^= 3.95, P < 0.05).

Concerning condom use, 41 (73.2%) of the urban resident MBs had condoms in MSM experiences over the past six months, whereas 15 (50.0%) of the rural residents had done the same (X^2 ^= 4.64, P < 0.05).

Differences between urban and rural MBs continued concerning type of typical client. Twenty of urban MBs (35.7%) had one regular client in a private house, while only 4 (13.33%) of the rural participants had one exclusive client (X^2 ^= 4.86, P < 0.05). In contrast, 14 (25.0%) urban MBs reported having random clients, while 17 rural MBs (56.7%) reported having random partners (X^2 ^= 7.74, P < 0.01).

## Discussion

The basic demographic and behavioral profile of money boys in Jilin Province is similar to that found in studies of other Chinese municipalities [3,12,19]. Most are young, and highly prone to risk behavior. As elsewhere, a substantial number of MBs in Jilin Province are married, and twenty-percent of the MBs also report having used a female commercial sex worker. It is becoming quite clear that wherever there is MB activity, there is also going to be a rather sizable portion who will serve as potential bridges for HIV to enter the broader heterosexual population [4].

However, the present data also make some interesting departures from previous investigations. Though most of the Jilin MBs maintained a characteristically high number of clients, some with over 20 reported sexual liaisons, the average number of clients for the group was relatively low at 4. This suggests that, especially in light of regional behavior variations, male sex work may vary from intense to only sporadic, yet all such behaviors must be addressed in an effective intervention strategy. In addition, 27.91 percent of the participants had only one exclusive client, and engaged in sexual activity in a private house. This percentage of MBs with exclusive clients may skew the average number of clients for the group, and account for the relatively low mean. None of these individuals were actually "kept" in the home, but only used the residence as a private venue. Intuitively, one might expect that the behavioral dynamics of such exclusive encounters, as well as the most effective health intervention strategy, would differ for this group. For example, Mi, et al., found that consistent condom use was associated with having regular sex partners [4]. Jilin MBs with regular or exclusive clients require further analysis as to how their behavior differs, or not, from those with random clients. As well, more emphasis needs to be placed on variations of behavior based upon sites in which respective MBs tend to work (i.e., private residences, bath houses, bars, the internet, etc.) [4,15].

Earlier analyses emphasized the propensity of male migrants to fall into MB activity. When current participants were divided into urban and rural categories, the majority were registered urban residents from Changchun or Jilin City. This supports the contention of Zhang and Chu [7] that HIV transmission dynamics in MSM/MB communities will vary according to "geographic and social elements." These preliminary data suggest that migrants may not necessarily be at the heart of money boy activity in Jilin Province. This finding is potentially important, since there is always a danger of increased social discrimination when one specific subgroup within a population is singled out as the source of a social problem. Nevertheless, rural migrants do have lower levels of HIV transmission knowledge, use condoms less frequently, and have more random commercial clients than their urban counterparts. As such, this subgroup demands continued scrutiny.

The concept of "economic necessity," while an important concept and highly relevant in areas in which MB activity is dominated by males from the floating population, seemed to have less relevance among the Jilin participants. None of the participants self-reported as heterosexual and engaging in MB activity to subsist. Most were gainfully employed and received a livable income by regional standards. While those participants with very high levels of clients would have been able to better supplement their regular income through MB activity, the average number of clients reported (4) would not be a source of substantial income unless there were very high levels of sexual activity. The average price of either oral or anal sex services in the province is only 100-200 yuan (approximately US$14.00-$28.00). However, frequency of sexual activity was not addressed in these data, nor was the concept of "underemployment" that might drive some to commercial sex activity. Finding that behavior is not driven by subsistence does not necessarily mean that economics does not contribute. Much more precision should be built into future studies in determining to what degree income motivates behavior.

Most prior studies have also found a poor level of understanding regarding basic HIV/AIDS knowledge, as well as a correlation between education and such knowledge [18,19]. Jilin MBs actually scored quite well on basic transmission knowledge and prevention, as well as knowledge of voluntary testing and counseling services now found throughout the country. No association, however, was discovered between educational level and HIV knowledge, as has been found previously [19].

Regardless of education, Jilin MBs seemed well aware of basic HIV transmission principles. Unfortunately, this knowledge did not translate well into positive behavioral change. None of the 86 participants reported always using a condom, and only 14 had used VCT services. Clearly, "mere" knowledge is not enough for this group. This finding adds support to a recent and thorough analysis of MBs in Shenzhen, in which it was found that condom use was low, even in the face of high HIV-related knowledge and knowledge of sero-status [14].

## Conclusion

Two primary caveats must be kept in mind when reading this report of the initial questioning of money boys in Jilin Province. First, the size of the convenience sample is small. However, the number of participants represents all MBs that could be located at this juncture of breaking into the Jilin Province community. The number of identified participants was impacted by the exceedingly covert nature of MB activity in Jilin Province, as well as the reach of the assisting NGO. Participants, nevertheless, fit nicely into previously established demographic characteristics (age, education, etc.) of MBs in other studies. As such, they may be said to be typical of the broader MB population from which they are drawn, though simply representing a convenience subgroup within it. As such, we do not contend that the sample is representative of the broader Jilin money boy population. We do contend that it provides a good baseline for future analysis, and keeps us mindful of the fact articulated by Zhang and Chu [7] that MB behavior may vary greatly, depending upon local circumstances and practices.

Second, there is the potential for selection bias in the sample, since all participants were located through contact facilitators associated with local MSM non-governmental organizations. The dominance of urban MBs is the sample may be an artifact of this selection method. Perhaps urban MBs are more likely to align themselves with a local organization than those coming from outside the urban area. As such, the dominance of urban participants in the survey, as well as their behavior when contrasted to rural participants, must be carefully reinvestigated in future studies in the province.

This first look at MBs in Jilin Province suggests that male commercial sex work *may not *necessarily be driven by migrants, though migrants do seem to be more prone to riskier commercial sex and more random sexual contacts than their urban counterparts. Money boys *may *possess a relatively high level of HIV/AIDS transmission knowledge, yet such knowledge is not reflected in the adoption of safer sex practices. Economic subsistence *may not *always be the primary driving factor in male commercial sex work. A blanket explanation of money boy activity, isolated from local circumstances and practices, is not in the offing.

Clearly, much more investigation needs to be conducted in the province before a more definitive picture of money boy behavior is obtained. Many questions are suggested in this report that await investigation, especially related to urban-rural differences in behavior, factors preventing behavioral change in light of good transmission and control knowledge, the low number of commercial partners relative to other studies, variability of condom use between non-commercial and commercial partners, and possible variations in behavior between MBs in different commercial venues. This exploratory look will allow us to ask the right questions as our analysis continues. One thing, however, is becoming clear as we work to develop a more definitive understanding of male commercial sex work in the province. Such activity can be ignored only at the peril of substantially reducing the effectiveness of broader HIV/AIDS prevention and control efforts in Jilin Province.

## Competing interests

The authors declare that they have no competing interests.

## Authors' contributions

MX participated in the development of the methodology, collected data, analyzed data, and developed the manuscript. AA participated in the development of the methodology, analyzed the data, and developed the manuscript. LZ participated in the development of the methodology, and collected and analyzed data. WL, GW, LZ, JH, and CY participated in the development of the methodology and collected data. All authors read and approved the final manuscript.

## Acknowledgements

This research was jointly funded by the Chinese Social Mobilization Project (2007) and the China-MSD AIDS Cooperation Project (2008). Funding bodies played no direct role in the research process.

## References

[B1] The Associated Press, International News AIDS becomes China's deadliest 2009 http://www.sfgate.com Accessed 18 February 2009

[B2] UNAIDS China Fact Sheet 2009 http://www.unaids.org/en/CountryResponses/Countries/China.asp Accessed 5 January 2010

[B3] Choi KH Gibson D Han L Yaqui G High levels of unprotected sex with men and women among men who have sex with men: a potential bridge of HIV transmission in Beijing, China AIDS education and Prevention 2004 16 1 19 30 10.1521/aeap.16.1.19.27721 15058708

[B4] Mi G Wu Z Zhang B Zhang H Survey of HIV/AIDS-related high risk behaviors among male sex workers in two cities in China AIDS 2007 21 S67 S72 10.1097/01.aids.0000304699.85379.32 18172394

[B5] Guo L Zhang L Jin Q Meta analysis: prevalence of HIV infection and syphilis among MSM in China Sexually Transmitted Infections 2009 85 354 358 10.1136/sti.2008.034702 19351623

[B6] Lau JTF Wang M Wong HN Tsiu HY Kia M Cheng F Zhang Y Su X Wang N Prevalence of bisexual behaviors among men who have sex with men (MSM) in China and association between condom use in MSM and heterosexual behaviors Sexually Transmitted Diseases 2008 35 406 413 10.1097/OLQ.0b013e318164467f 18362864

[B7] Zhang BC Chu QS MSM and HIV/AIDS in the People's Republic of China Cell Research 2005 15 858 864 10.1038/sj.cr.7290359 16354560 PMC1791010

[B8] Hong FC Zhou H Cai Y Pan P Feng T Liu X Chen X Prevalence of syphilis and HIV infections among men who have sex with men from different settings in Shenzhen, China: implications for HIV/STD surveillance Sexually Transmitted Infections 2009 85 42 44 10.1136/sti.2008.031682 18653567

[B9] UNAIDS China to tackle HIV infections amongst MSM 2009 http://unaids.org/en/KnowledgeCentre/Resources/FeatureStories/archive/2009/20090116_MSMAsia.asp Accessed 18 February 2009

[B10] Chan WT A Sourcebook in Chinese Philosophy 1963 Princeton: Princeton University Press

[B11] Aegis-Reuters Sex taboos hamper safety message for gay Chinese 2006 http://www.aegis.com/news/re/2006/re060830.html Accessed 13 August 2006

[B12] Liu HJ Liu H Cai Y Rhodes A Hong F Money boys, HIV risks, and the associations between norms and safer sex: a respondent-driven sampling study in Shenzhen, China AIDS and Behavior 2009 13 652 662 10.1007/s10461-008-9475-0 18841459

[B13] China Daily 4 members of a male sex ring jailed 2008 http://chinadaily.com Accessed 15 January 2009

[B14] Jeffreys E Querying queer theory: debating male-male prostitution in the Chinese media Critical Asian Studies 2007 39 1 151 175 10.1080/14672710601171772

[B15] Cai WD Zhao J Zhao Jk Fisher R Feng YJ Liu J McFarland W Gan YX Yang ZR Zhang Y Tan JG Wang XR He ML Cheng JQ Chen L HIV prevalence and related risk factors among male sex workers in Shenzhen, China--results from a time-location-sampling survey Sexually Transmitted Infections 2009 in press doi:10.1136/sti2009.037440 19854703 10.1136/sti.2009.037440

[B16] Zhang B Liu D Li X Hu T A survey of men who have sex with me in China American Journal of Public Health 2000 90 2 1949 1950 10.2105/AJPH.90.12.1949 11111277 PMC1446424

[B17] Kong TSK Risk factors affecting condom use among male sex workers who serve men in China: a qualitative study Sexually Transmitted Infections 2008 84 444 448 10.1136/sti.2008.030650 19028944

[B18] He N Wong FY Huang JZ Fu C Smith BD Young D Jiang Q HIV risk among two types of male migrants in Shanghai, China, money boys vs. general male migrants AIDS 2007 21 Suppl 8 573 579 10.1097/01.aids.0000304700.85379.f3 18172395

[B19] Wong FY Huang Z He N Smith BD Ding Y Fu C Young D HIV risks among gay and non-gay-identified migrant money boys in Shanghai, China AIDS Care 2008 20 2 170 180 10.1080/09540120701534707 18293125

[B20] Higgins DL O'Reilly K Tashima N Crain C Beeker C Goldbaum G Elifson CS Galavotti C Guenter-Grey C Using formative research to lay the foundation for community level HIV prevention efforts: an example from the AIDS Community Development Projects Public Health Reports 1996 111 28 35 8862154 PMC1382040

[B21] Choi KH Diehl E Yaqi G Qu S Mandel J High HIV risk but inadequate prevention services for men in China who have sex with men: an ethnographic study AIDS and Behavior 2002 6 255 266 10.1023/A:1019895909291

